# A Mindfulness Program Addressing Sleep Quality and Stress: Transition to a Telehealth Format for Higher Education Students During COVID-19

**DOI:** 10.5195/ijt.2022.6439

**Published:** 2022-06-03

**Authors:** Sara Benham, Nabila Enam, Samanvita Ivaturi

**Affiliations:** 1 Moravian University, Department of Rehabilitation Sciences, Bethlehem, PA, USA; 2 University of the Sciences, Department of Occupational Therapy, Philadelphia, PA, USA; 3 Hackensack Meridian Health Nursing and Rehabilitation, Shrewsbury, NJ, USA

**Keywords:** Life stress, Occupational therapy, Sleep habits, Students, Telehealth

## Abstract

Many higher education students report sleep problems, further exemplified along with stress at the onset of the COVID-19 pandemic. Promising evidence supports the use of mindfulness programming, although synchronous telehealth sessions have not been adequately examined. This exploratory eight-session telehealth mindfulness program utilized a pretest-posttest quantitative design to examine changes in sleep quality and perceived stress for 16 higher education students enrolled at a health professions-focused university. Sleep quality changes were measured using the Pittsburgh Sleep Quality Index (Z=-3.234, p=0.0012, d=-0.808) and perceived stress with the Perceived Stress Scale (Z=-3.102, p=0.0019, d=-0.776), both of which were significant. The results suggest that synchronous mindfulness programming delivered via telehealth has the potential to improve sleep quality and perceived stress in students, however, future studies should consider the use of objective measurements of sleep duration and quality, and a control group.

Sleep is defined as an altered state of consciousness where the mind and body repair and recharge themselves, yet the lack of sleep is a national issue as over a third of adults in the United States report difficulty with sleep (Liu et al., 2013; Tester & Foss, 2018). The young adult population, ages 18-25 years, is recommended to sleep seven to nine hours per night; however, approximately a third of this age group report less than seven hours of sleep a night (Centers for Disease Control and Prevention [CDC], 2017; Hirshkowitz et al., 2015). About 62% of students in higher education experience poor sleep quality and those that reported poor sleep quality also reported more problems with their physical and mental health (Becker et al., 2018; Lund et al., 2010). Young adults often participate in higher education resulting in decreased sleep due to new social opportunities, increased freedom, challenging coursework, and the added burden of financial stressors such as paying for tuition and rent (CDC, 2017; Hershkowitz et al., 2015; Hershner, 2015). These transitional life changes may also be related to mental health, particularly stress and anxiety (Amaral et al., 2018). There is a strong association between perceived stress levels, depression, and sleep quality, indicating a link between sleep quality and other health outcomes (Lemma et al., 2012).

Numerous studies suggest links between sleep quality and academic performance as students who reported poor sleep quality also reported lower GPAs (Lemma et al., 2015). Of particular concern are health professions students as the students prepare for healthcare professions that are associated with stress, anxiety, and decreased sleep (Shreffler et al., 2020). Among medical students, those who had average academic performance also reported poor sleep quality, while only a minority of excellent academic scorers reported poor sleep quality (Mirghani et al., 2015). Nursing students were found to have poor sleep quality resulting in decreased academic success, poor emotional status, and increased substance abuse, which may result from rigorous coursework and schedules contributing to reduced sleep quality (Yilmaz et al., 2017).

Moreover, relationships between sleep quality and emotional status or stress have been confirmed. Higher stress levels are significantly associated with poor sleep quality in first-year college students (Garret et al., 2017), and lower levels of stress are associated with more favorable sleep health (Benham, 2019). The COVID-19 pandemic onset further altered stress levels and everyday educational and social interactional routines (Singh & Singh, 2020). Early pandemic survey results indicated that 71-79% of students reported increased stress and anxiety due to the pandemic and 86% had increased disruptions to their sleep patterns (Son et al., 2020; Wang et al., 2020). Graduate and undergraduate students among seven countries during the pandemic mostly reported meeting the recommended sleep duration, but about 60% were poor sleepers based on the Pittsburgh Sleep Quality Index (PSQI), 40% experienced severe to moderate anxiety, and 85% experienced moderate to high levels of perceived stress, indicating relationships between poor sleep quality and increased anxiety and stress (Du et al., 2020). Comparing pre-pandemic student reports in 2019 to pandemic reports in 2020, students reported significantly greater sleep medication use, lower sleep efficiency, and disrupted sleep routines of later bedtimes and delayed wake times during the pandemic, as compared to pre-pandemic routines (Benham, 2021). However, some positive changes were observed as a more significant proportion of students obtained at least seven hours of sleep a night (Benham, 2021). This may indicate the resiliency of younger adults to adapt their lifestyle routines and their responsiveness to change as university-level policies and coursework delivery formats frequently were altered (Benham, 2021).

Many university-level changes were enacted at a rapid expansion. These included course content delivery shifts to online platforms and changes in the formats of institutional support programs available to students, including wellness programs. Mindfulness, as part of a sleep program, is becoming an increasingly utilized, evidence-based method for improving sleep within wellness programs (Friedrich & Schlarb, 2017). Mindfulness is described as the awareness that emerges through paying attention to purpose and presence, and nonjudgmentally unfolding experiences moment by moment (Barnhofer et al., 2009; Kabat-Zinn, 2003). As reported through systematic review findings, interventions integrating mindfulness are promising to address psychological well-being for adults across the lifespan (Eberth & Sedlmeier, 2012; Janssen et al., 2018). When considering this transition to digital or online service delivery formats, asynchronous modules versus live face-to-face telehealth sessions may produce inconsistent results. For an eight-session, pre-recorded web-based mindfulness program for young adults, high attrition was a major limitation (Mak et al., 2017), and similar findings of low compliance among adolescents have also been reported for asynchronous programming (Antonson et al., 2018), which suggests the need for more personalized content. Examining a randomized control trial of a two-week self-guided mindfulness program, no differences in sleep quality or perceived stress resulted between the intervention and control. However, in the review of an eight-week web-based program that included videoed modules, anonymous videoconferences, and discussion forums, there were reductions in perceived stress compared to the control group (Cavanagh et al., 2018). This may justify longer durations than two weeks, tailored interventions, and synchronous telehealth.

While there is an adequate amount of literature stating that sleep is essential, there is a lack of research that offers interventions to address sleep quality in health professions students. Additionally, examinations of the outcomes of the transition of mindfulness programming to telehealth adaptations during the COVID-19 pandemic have not been reported.

Occupational therapy service delivery includes the promotion of healthy sleep behaviors and quality sleep with the facilitation of habits, roles, and routines, as well as environmental adaptation (AOTA, 2020; Leland et al., 2014). A systematic review concluded that occupational therapy sleep interventions included assistive devices, mindfulness activities, cognitive-behavioral therapy, and lifestyle modifications (Ho & Siu, 2018). In consideration of the transition to an online format for mindfulness and sleep interventions, occupational therapy practitioners use telehealth to help clients improve skills, use assistive technology or adaptive devices, modify environments and contexts where daily occupations occur, and create and enhance habits and routines (Cason et al., 2013). However, further evidence should be explored regarding telehealth delivery for mindfulness practices for sleep and stress management, as little to no literature exists for interventions for students preparing for health professions. A mindfulness meditation program presents a potential solution and guides this study's primary research question: After eight sessions of mindfulness meditation practice through telehealth delivery, is there a change in sleep quality as measured by the Pittsburgh Sleep Quality Index (PSQI) for higher education students enrolled at a health professions-focused university? The secondary question is: After eight sessions of mindfulness meditation practice, is there a change in perceived stress as measured by the Perceived Stress Scale (PSS)?

## METHODS

This study utilized an exploratory, one-group pretest-posttest design to examine the effects of a mindfulness meditation program on sleep quality and perceived stress in students in higher education. The study procedures were piloted via a telehealth platform as investigators explored a previously established in-person mindfulness program, to track sleep quality and perceived stress changes for participants. The setting was a private, urban-based East Coast university, focusing on physical sciences and professional health sciences curricula. Supportive services offered by the university, such as academic advising, counseling, and financial aid meetings, were largely offered virtually due to the COVID-19 pandemic. The mindfulness meditation program consisted of eight virtual sessions over the course of twelve weeks.

### PARTICIPANTS

After obtaining IRB approval, participants were recruited using non-probability convenience sampling of undergraduate and graduate students enrolled during the 2020-2021 academic year. Emails were sent to student organization representatives and flyers were posted around campus locations on bulletin boards approved for student affairs, which included high-traffic areas such as the library, the dining hall, the recreation center, and outside of classrooms. Student organization representatives were given information about the study to pass along to interested members. Participants were eligible for the study if they were full-time or part-time students, were 18 years or older, had access to an internet connection and an electronic device, and were able to comprehend and speak English. Exclusion criteria were any potential participants that were not enrolled as students at the university and not having access to Zoom.

### PROCEDURES

The mindfulness meditation program consisted of eight virtual sessions via Zoom (Zoom Video Communications, Inc., https://zoom.us) held during the Spring 2021 semester. Participants could attend sessions up to two times a week; thus, the duration could be a minimum of four weeks, however all sessions were required to be completed at a maximum of twelve weeks, to allow for flexibility of unforeseen absences such as illnesses. Sessions were 30 minutes long, allotting for a five-minute introduction, 15 minutes of guided mindfulness meditation, and 10 minutes of discussion. Trained student researchers led the guided mindfulness sessions and used the same verbal script for every session, adapted from two publicly available scripts (Cuncic, 2020; The Mindful Movement, 2018) with gentle music playing in the background. Sessions were offered three times a day from Monday to Friday. Participants attended one to two sessions per week until the completion of the eight sessions. Participants were required to register for the sessions and were able to schedule alternate sessions. Discussions held during sessions included educating participants on the uses of mindfulness meditation outside of the study and providing them with resources to incorporate mindfulness into daily life activities, such as prior to exams, preparations for internship interviews, and other stressful moments shared during the discussions. Participant questions about mindfulness and the program were answered during this time. The duration of eight sessions was chosen based on the previous effectiveness in reducing perceived stress with in four to eight weeks through virtual approaches (Ahmad et al., 2020) while considering the flexibility of offering times required for the higher education student.

### MEASUREMENT

The outcome measures were administered immediately prior to the first intervention session and immediately after the final intervention session. Participants completed the outcome measurement assessments, along with the demographics survey, through Qualtrics® (Qualtrics, Provo, UT). The PSQI is a self-report index that assesses sleep quality and distinguishes “good” sleepers from “poor” sleepers (Buysse et al., 1989). This measurement asks questions about the participant's sleep in the last month and consists of 19 self-report questions with summed scores used to obtain a global PSQI score. Questions include topics of subjective sleep quality, sleep latency, sleep duration, and frequency and severity of sleep-related difficulties. The range of the global PSQI scores is 0 to 21, with higher scores indicating poorer sleep quality. A cut-off of a global PSQI score of greater than 5 indicates poor sleep quality (Buysse et al., 1989). The PSQI has been shown to have good test-retest reliability (r=0.85), with Cronbach's α internal consistency among the seven components as 0.83 (Buysse et al., 1989). In a sample of students at a university in the United States, the PSQI showed moderate convergent validity with other assessments that measure aspects of sleep, such as the sleep diary (r=0.53), the Insomnia Severity Index (0.63), and the Multidimensional Fatigue Inventory-General Fatigue Scale (r=0.44) (Dietch et al., 2016).

The PSS is a self-report scale that assesses perceived stress and consists of ten questions with a five-point Likert-type scale to record responses (Cohen et al., 1983). Responses can range from 0, which indicates never, to 4, which indicates very often. Questions are general, asking about stressors the participant may have experienced in the last month. Questions are either positively phrased (e.g. In the last month, how often have you felt that things were going your way?) or negatively phrased (e.g. In the last month, how often have you felt upset because of things that happened unexpectedly?). The scores of the positively phrased questions numbered 4, 5, 7, and 8 are reversed and then the scores of each question are added together to obtain a total score (Cohen & Williamson, 1988). The total score ranges from 0 to 40, with a scores of 0 to 13 indicating low stress, scores of 14-26 indicating moderate stress, and scores of 27 to 40 indicating high stress (Eskildsen et al., 2015). Internal consistency for the PSS is moderate (Cronbach's α=0.78) (Cohen & Williamson, 1988) and test-retest reliability is moderate to high (r=0.55-0.85) (Lee, 2012s).

### DATA ANALYSIS

IBM SPSS Statistics package was used for all analyses (SPSS Version 26.0; IBM Corp., Armonk, NY). Descriptive analyses were used to report the participant demographics. The within-group results of the PSQI and the PSS were analyzed using the Wilcoxon signed rank test and type I error was set at 5%. We hypothesized an improvement at posttest with significant differences when compared to pretest. Cohen's d was used as the estimate for effect sizes. Results were interpreted as <0.2, trivial/negligible effect; 0.2-0.4, small effect; 0.5-0.8, moderate effect; >0.8, large effect (Cohen, 1998). In addition, we calculated the median and interquartile ranges (IQR) for each PSS individual question pretest to posttest, as well as Wilcoxon analyses, to provide insight into the possible stressors during the pandemic.

Based on previous literature, we utilized anticipated effect sizes between 0.60 and 0.76 (Greeson et al., 2014; Huberty et al., 2019). Through the power analysis calculation planning for power at 80% and a one-tailed analysis, this indicated a range of participants, then increased to account for a potential 20% attrition rate, to aim to enroll from 17 to 20 participants.

## RESULTS

During the recruitment phase, 21 students enrolled in the study by completing the informed consent procedures. One participant did not respond to further email communication and did not complete the pre-test measures. Four students withdrew from the study, with loss of contact. The remaining 16 students completed the eight sessions of the program and were included in the final data analysis. Demographic data of the participants are included in Table 1, with 93.75% female participants and a mean age of 22.44 (2.45). Race, majors, credit load, and employment status data of the participants are also included.

**Table 1:** Participant Demographics (n = 16)

Demographic	n (%) or Mean (SD)
Gender	
Female	15 (93.75%)
Male	1 (6.25%)
Race	
Asian	7 (43.75%)
Black or African American	1 (6.25%)
White	8 (50.00%)
Major	
Health Science	11 (68.75%)
Pharmaceutical Sciences	1 (6.25%)
Other	4 (25.00%)
Employment Status	
Employed	10 (62.50%)
Not Employed	6 (37.50%)
Age	22.44 (2.45)
Credit Load	16.25 (2.18)

*Note:* SD= standard deviation

To address the primary research question, the results of the pretest to posttest revealed significant changes of the outcome measure of the global PSQI mean score, of an overall mean change of a decrease of 2.82 (0.34), *Z*=-3.234, *p*=0.0012, *d*=-0.808 (see Table 2). The significant decrease in global scores indicates an improvement in sleep quality after the completion of the program, with a large effect following Cohen's guidelines (1998).

**Table 2:** Outcome Measures Data (n =16)

	Pretest Mean (SD)	Posttest Mean (SD)	Change Mean (SD)	*Z*	*p*	*d*
PSQI Global Score	7.88 (2.42)	5.06 (2.08)	-2.82 (0.34)	-3.234	0.0012^*^	-0.808
PSS Total Score	19.63 (5.03)	13.44 (4.34)	-6.19 (0.69)	-3.102	0.0019^*^	-0.776

*Note:* PSQI=Pittsburgh Sleep Quality Index, PSS=Perceived Stress Scale;

* *p* ≤ 0.05; decreases in scores of both the PSQI and the PSS indicate improvements in outcomes.

The data analyzed from the PSS addresses the secondary research question, also presented in Table 2, which shows a decrease in the mean change from pretest to posttest of 6.19 (0.69), which is statistically significant at *Z*=-3.102, *p*=0.0019, *d*=-0.776. This indicates that perceived stress experienced by participants after the mindfulness meditation program decreased, from pretest to posttest, with an effect size of -0.776 which is considered to be a medium effect (Cohen, 1998). Non-parametric Wilcoxon analyses were also utilized to determine changes from pretest to posttest of each PSS question scoring, which is presented in Table 3. The scores from 8 of the 10 questions (questions 2-4 and questions 6-10) may be interpreted as significant changes (see Table 3). These questions mainly asked participants regarding coping with external factors and other factors outside of their control (Cohen et al., 1983). Question 1, which asked participants how often they felt upset because of unexpected situations, did not show significant changes from pretest to posttest (*p*=0.141), as well as Question 5, which asked how often they felt things were going their way (*p*=0.059) (Cohen et al., 1983).

**Table 3:** Perceived Stress Scale Individual Questions (n =16)

		Median (IQR)	*p*
PSS Question	Pretest	Posttest	
1. In the last month, how often have you been upset because of something that happened unexpectedly?	2 (1.75, 3)	2 (1, 2)	0.141
2. In the last month, how often have you felt that you were unable to control the important things in your life?	2 (1.75, 3)	2 (1, 2)	0.038^**^
3. In the last month, how often have you felt nervous and “stressed”?	3 (2, 4)	2 (1.75, 3)	0.005^**^
4. In the last month, how often have you felt confident about your ability to handle your personal problems?^*^	2 (2, 2)	3 (2, 3)	0.033^**^
5. In the last month, how often have you felt that things were going your way?^*^	2 (2, 3)	3 (2, 3)	0.059
6. In the last month, how often have you found that you could not cope with all the things that you had to do?	2 (1, 2)	1 (0.75, 2)	0.031^**^
7. In the last month, how often have you been able to control irritations in your life?^*^	2 (2, 2.25)	3 (3, 3)	0.005^**^
8. In the last month, how often have you felt that you were on top of things?^*^	2 (2, 3)	3 (2, 3.25)	0.008^**^
9. In the last month, how often have you been angered because of things that were outside your control?	2 (1.75, 3)	1 (1, 2)	0.012^**^
10. In the last month, how often have you felt difficulties were piling up so high that you could not overcome them?	2 (1, 2)	1 (1, 1.25)	0.029^**^

*Note:* IQR = interquartile range

* positively-phrased questions are reverse-scored for PSS total scoring

** *p* ≤ 0.05

## DISCUSSION

The purpose of this study was to explore changes in sleep quality and perceived stress among students in higher education after their participation in telehealth mindfulness programming. The results of this pilot program revealed significant improvements in sleep quality and perceived stress from pretest to posttest, as the null hypothesis was rejected. While the results of the PSQI were significant, the posttest global mean score, 5.06, indicates that participants still experienced some level of poor sleep quality after the program, as the cut-off of greater than 5 indicates poor sleep, however, is close to the cut off of 5. Furthermore, the MCID value of 3 points was not met as the difference between the pretest global mean and posttest global mean scores was 2.82 (Backhaus, et al., 2002). Significant changes overall with a large effect size indicate the potential for this program to improve sleep quality. Additionally, as the semester progressed, participants' academic workload increased, which might have affected how participants rated their sleep when completing the posttest.

The PSS also showed significant changes with a medium effect size in perceived stress after completion of the mindfulness program, therefore it may be interpreted that the program potentially contributed to decreased reports of stress.

The pretest total PSS mean score of 19.63 (5.03) may be interpreted that as a group, prior to the mindfulness meditation program participants experienced *moderate stress*. The posttest total PSS mean score of 13.44 (4.34) indicates that after the mindfulness meditation program, that the group of participants experienced a decrease in stress however not quite meeting the cut-off of 13 to be considered as *low stress* (Eskilds en et al., 2015). The MCID for the PSS is 11 points (Eskildsen et al., 2015), which was not met as the difference between the pretest and posttest mean score was 6.19. Further examination of individual questions may provide insight. The scores of questions 2-4 and 6-10 showed significant changes. These questions, both positively and negatively phrased, mainly asked about how the participants felt they could cope with external factors or how often they were able to control irritations or the important things in their life. The findings may suggest that after completing the mindfulness meditation program, the participants were better able to manage the external factors of their lives that they might not have control over. The PSS did not show significant improvements in two of the PSS questions, question 1 and question 5. Question 1 asked participants how often they felt upset because of something that happened unexpectedly, and question 5 asked how often they felt that things were going their way. Many different aspects could have contributed to how participants rated themselves when answering these questions. Due to COVID-19 university policies, students were required to alternate between in-person and online instruction formats for courses throughout the semester, student organization and club meeting alternative modifications, and unexpected scheduling changes throughout the semester. For example at this university, the week-long spring break from classes was cancelled during this semester.

The results of this study are consistent with the findings of several other studies with similar populations, inclusive of student-athletes, nursing students, and other students in higher education (Huberty et al., 2019; Li et al, 2018; Spadaro & Hunker, 2016). With the unexpected shift of programming requirements to transition to utilize online formats, the data suggest that the telehealth mindfulness program findings are consistent with current literature regarding mindfulness and the effects on sleep quality and perceived stress. These findings of this study's delivery of telehealth programming provide a potential solution to address the poor sleep quality and increased stress often associated with higher education, utilized as a potential intervention that may be associated with performance and satisfaction in other daily activities such as academic performance (Lemma et al., 2015; Mirghani et al., 2015).

This study was conducted due to a lack of literature addressing sleep problems in higher education students, especially related to occupational therapy interventions surrounding sleep quality and perceived stress in higher education students. The results support providing virtual mindfulness programming at higher education institutions and inform and guide occupational therapy practice as it provides a potential intervention to be used with this population. Occupational therapy practitioners are able to address sleep management with their clients and can facilitate improvements in sleep quality through habituation, consequently improving engagement in other occupations. As administered via a telehealth adaptation, the intervention has the potential to decrease stress, therefore improving one's overall health, wellness, and quality of life. This virtual mindfulness program was a novel approach to provide sleep interventions for students in higher education and may be an effective intervention to use via telehealth with clients experiencing poor sleep and high stress. The results of this study suggest that mindfulness practiced through virtual means may be beneficial for students in higher education. Furthermore, it provides occupational therapy practitioners working in the emerging practice area of higher education and health and wellness with valuable information.

### LIMITATIONS

The demographics survey questions did not ask participants' university status. For example, if they were a full-time student or a part-time student, an undergraduate student, or a graduate student. This information would have been helpful when analyzing PSQI and PSS scores as the researchers could have compared the data from each group and reported on differences in scores between the groups. Furthermore, the study participants' gender majority was female, so PSQI and PSS scores between males, females, and nonbinary participants were not examined. This information would have been helpful when analyzing data to compare differences between these groups. Self-selection bias among females may exist so random sampling may be warranted for future research. In addition, this study was underpowered, as the sample size aim was between 17 and 20. The high attrition rate of five participants withdrawing from the study was unexpected, however, provides insight for future study planning to potentially over-enroll in anticipation of dropouts, although dropouts may have been related to pandemic-related stressors.

In the future, studies should expand with a larger sample size to recruit diverse students and increase generalizability. A control group of asynchronous education modules is the next step to attribute the effects of the telehealth program directly to the observed outcome measures. Also, an objective sleep quality measurement will reduce self-reporting bias, along with recording measurements of sleep duration, with the use of accelerometry. Future research should include a control group of asynchronous education modules to attribute the effects of the results directly to the synchronous intervention. Furthermore, providing participants with more time slots to attend sessions throughout the day may provide more ease and flexibility contributing to student participation retention.

## CONCLUSIONS

Results from the outcome measures revealed that there were significant improvements in sleep quality and perceived stress. This pilot program suggests that mindfulness can potentially be utilized as an intervention for sleep management, as well as stress management in students in higher education delivered via telehealth. From an occupational therapy framework, sleep and stress affect performance and satisfaction of other daily activities, addressing these factors may help improve other occupations in which clients engage. These practices can be utilized in higher education programming to address the poor sleep quality and high stress that students in higher education often experience. Occupational therapy practitioners may be consulted in addressing sleep management and can potentially use virtual mindfulness meditation to improve sleep quality and stress, specifically incorporating telehealth sleep interventions into practice.
